# MEMS Inertial Sensors-Based Multi-Loop Control Enhanced by Disturbance Observation and Compensation for Fast Steering Mirror System

**DOI:** 10.3390/s16111920

**Published:** 2016-11-15

**Authors:** Chao Deng, Yao Mao, Ge Ren

**Affiliations:** 1 Institute of Optics and Electronics, Chinese Academy of Science, Chengdu 610209, China; chaosir1991@gmail.com (C.D.); renge@ioe.ac.cn (G.R.); 2 Key Laboratory of Optical Engineering, Chinese Academy of Sciences, Chengdu 610209, China; 3 University of Chinese Academy of Science, Beijing 100039, China

**Keywords:** MEMS inertial sensors, disturbance observation and compensation, multi-loop feedback control, light of sight stabilization

## Abstract

In this paper, an approach to improve the disturbance suppression performance of a fast steering mirror (FSM) tracking control system based on a charge-coupled device (CCD) and micro-electro-mechanical system (MEMS) inertial sensors is proposed. The disturbance observation and compensation (DOC) control method is recommended to enhance the classical multi-loop feedback control (MFC) for line-of-sight (LOS) stabilization in the FSM system. MEMS accelerometers and gyroscopes have been used in the FSM system tentatively to implement MFC instead of fiber-optic gyroscopes (FOG) because of its smaller, lighter, cheaper features and gradually improved performance. However, the stabilization performance of FSM is still suffering a large number of mechanical resonances and time delay induced by a low CCD sampling rate, which causes insufficient error attenuation when suffering uncertain disturbances. Thus, in order to make further improvements on the stabilization performance, a cascaded MFC enhanced by DOC method is proposed. The sensitivity of this method shows the significant improvement of the conventional MFC system. Simultaneously, the analysis of stabilization accuracy is also presented. A series of comparative experimental results demonstrate the disturbance suppression performance of the FSM control system based on the MEMS inertial sensors can be effectively improved by the proposed approach.

## 1. Introduction

The fast steering mirrors (FSMs) play a critical role in optical fine tracking control systems, such as for adaptive optics, long-distance laser communication, line-of-sight (LOS) stabilization, which are increasingly mounted on vehicles, airplanes, spacecraft and other moving platforms [1,2,3,4]. In the classical FSM control system, the fiber-optic gyroscopes (FOGs) and charge-coupled devices (CCDs) are generally used to implement a dual closed-loop control to stabilize LOS [5,6]. High closed-loop bandwidth facilitates good closed-loop performance. However, the control bandwidth is limited mainly by mechanical resonances, time delay, and also the sensors’ noise.

With the development of the MEMS industry in recent years, the performance of micro-electro-mechanical system (MEMS) inertial sensors, including accelerometers and gyroscopes, have been rapidly improved [7,8]. Considering the installation position of the sensors limited to the narrow spaces of the reverse side of the mirror, both MEMS accelerometer and gyroscope can be mounted on the frame of FSM due to the relatively small size and low weight [7,8,9,10]. Usually, the bandwidth of MEMS gyroscopes with low noise is generally less than 100 Hz, which limits the bandwidth of the velocity closed-loop and the disturbance suppression ability; nevertheless, the bandwidth of MEMS accelerometers exceeds 800 Hz. Thus, the high bandwidth acceleration feedback loop implemented by two linear MEMS accelerometers can be used to improve the disturbance suppression ability of FSM control system [7,9].

The acceleration feedback control (AFC) is a kind of high-precision robust control method to form multi-loop feedback control (MFC), which has been widely used in some high precision systems such as telescopes, missiles and robots [11,12,13]. In FSM systems, the acceleration closed loop can improve the stiffness of the system. In previous works, some researchers focused on how to design the acceleration controller because of the quadratic differential in the FSM transfer model. To avoid the saturation of double integration, Tang designed the controller as a band-pass filter and combined a CCD and accelerometers to implement dual closed-loop control [14]. Tian used a CCD, gyroscopes and accelerometers to implement a three-closed-loop control model, and a lag controller was used to accomplish acceleration control, which improved the disturbance suppression performance powerfully [9,15]. But the error rejection ability of the FSM system is still not adequate when suffering some uncertain disturbances, because of the mechanical resonances, CCD time delay and the drift noise in MEMS inertial sensors [14].

In this paper, to further enhance the stabilization performance, we proposed a new FSM stabilization control method, which combines the MFC with the disturbance observation and compensation (DOC) method. The MFC can be used to improve the stiffness of the FSM, and the DOC is utilized to estimate and compensate the outer base disturbance [16,17,18,19,20]. The compensating precision of DOC mostly depends on the mathematical model of the controlled plant [16,17]. In FSM control systems, the acceleration response mathematical model can be achieved in high accuracy by a spectral analyzer, which is conducive to DOC fulfillment in practical systems. As a consequence, a detailed introduction to the model of the FSM is presented in Section 2. And then the theory analysis and the controller design are shown in Section 3 and Section 4. The verification experiment is discussed in Section 5. Concluding remarks are presented in Section 6.

## 2. FSM System Control Model

**Figure 1:**
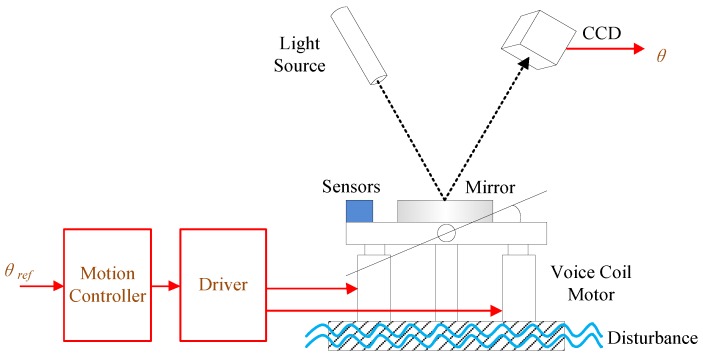
The structure of the fast steering mirror (FSM) control system.

A FSM is generally defined as a mirror mounted to a flexure support system and driven by actuators [21,22]. The schematic structure of the FSM control system is illustrated in Figure 1. The voice coil motors are used to drive the FSM platform to achieve the stabilization of LOS [22].

The mechanical part of the FSM is a typical resonance element and the voice coil motors are a typical first-order inertial element in the mathematical model. Therefore, the FSM acceleration controlled model including a quadratic differential can be expressed as follows [9,15,21,23]:

(1) $$G_{a}(s)=\frac{\ddot{\theta }(s)}{U(s)}=\frac{K}{\frac{s^{2}}{\varpi_{n}{}^{2}}+\frac{2\xi }{\varpi_{n}}s+1}\cdot \frac{s^{2}}{T_{e}s+1}$$

The control strategy of FSM goes into particulars as follows.

### 2.1. Multi-Loop Feedback Control (MFC)

**Figure 2:**
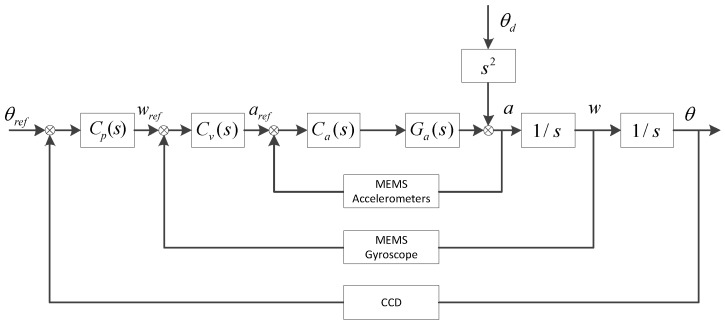
Classical multi-loop feedback control (MFC) structure. $G_{a}(s)$ is the controlled plant, $C_{a}(s)$ is the acceleration controller, $C_{v}(s)$ is the velocity controller, $C_{p}(s)$ is the position controller, $\theta_{d}$ is the outer disturbance angle ,and $\theta_{ref}$ is the given target position.

The classical stabilization control structure of multi-loop feedback control in FSM system is shown in Figure 2 [9,15].

According to the control structure, it is easy to obtain,

(2) $$\theta =\frac{C_{p}C_{v}C_{a}G_{a}\frac{1}{s^{2}}}{1+C_{a}G_{a}+C_{v}C_{a}G_{a}\frac{1}{s}+C_{p}C_{v}C_{a}G_{a}\frac{1}{s^{2}}}\theta_{ref}+\frac{1}{1+C_{a}G_{a}+C_{v}C_{a}G_{a}\frac{1}{s}+C_{p}C_{v}C_{a}G_{a}\frac{1}{s^{2}}}\theta_{d}$$

As the purpose of this work is not the tracking problem, but the stabilization of FSM, the given target position $\theta_{ref}=0$. The disturbance suppression of FSM with the classic three closed-loops method is given in Equation (3):

(3) $$E_{MFC}=\frac{\theta }{\theta_{d}}=\frac{1}{1+C_{a}G_{a}+C_{v}C_{a}G_{a}\frac{1}{s}+C_{p}C_{v}C_{a}G_{a}\frac{1}{s^{2}}}$$

Therefore, it can be seen that the performance of MFC depends on the entire three closed-loops. An appropriate controllers design can obtain the minimum $E_{MFC}$, which means the least effect from the disturbance $\theta_{d}$.

### 2.2. Multi-Loop Feedback Control with Disturbance Observation and Compensation (MFC-DOC)

**Figure 3:**
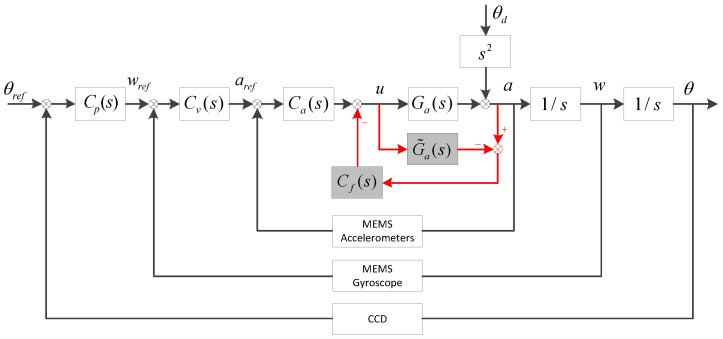
The multi-loop feedback control with disturbance observation and compensation (MFC-DOC) structure. ${\tilde{G}}_{a}(s)$ is the approximate model of the controlled plant, which can be obtained by fitting the measured transfer model, and $C_{f}(s)$ is the disturbance compensation controller.

The modified structure of MFC-DOC is shown in Figure 3.

The closed-loop acceleration is given in Equation (4):

(4) $$\begin{array}{l} a=uG_{a}+s^{2}\theta_{d}\\ u=(a_{ref}-a)C_{a}-(a-u{\tilde{G}}_{a})C_{f} \end{array}$$

As the element u is a substitutable factor, after substitution, it is easy to obtain:

(5) $$a-s^{2}\theta_{d}=(a_{ref}-a)C_{a}G_{a}-[aC_{f}G_{a}-(a-s^{2}\theta_{d}){\tilde{G}}_{a}C_{f}]$$

(6) $$[1+C_{a}G_{a}+(G_{a}-{\tilde{G}}_{a})C_{f}]a=C_{a}G_{a}a_{ref}+(1-{\tilde{G}}_{a}C_{f})s^{2}\theta_{d}$$

So,

(7) $$a=\frac{C_{a}G_{a}}{1+C_{a}G_{a}+(G_{a}-{\tilde{G}}_{a})C_{f}}a_{ref}+\frac{(1-{\tilde{G}}_{a}C_{f})s^{2}}{1+C_{a}G_{a}+(G_{a}-{\tilde{G}}_{a})C_{f}}\theta_{d}$$

According to the control structure, it is easy to get:

(8) $$a_{ref}=C_{v}(w_{ref}-w)$$

Then, the closed-loop velocity transfer function can be given:

(9) $$w=\frac{C_{v}C_{a}G_{a}\frac{1}{s}}{1+C_{a}G_{a}+(G_{a}-{\tilde{G}}_{a})C_{f}+C_{v}C_{a}G_{a}\frac{1}{s}}w_{ref}+\frac{(1-{\tilde{G}}_{a}C_{f})s}{1+C_{a}G_{a}+(G_{a}-{\tilde{G}}_{a})C_{f}+C_{v}C_{a}G_{a}\frac{1}{s}}\theta_{d}$$

Similarly, the closed-loop angular position will be depicted as follows:

(10) $$\theta =\frac{C_{p}C_{v}C_{a}G_{a}\frac{1}{s^{2}}}{1+C_{a}G_{a}+(G_{a}-{\tilde{G}}_{a})C_{f}+C_{v}C_{a}G_{a}\frac{1}{s}+C_{p}C_{v}C_{a}G_{a}\frac{1}{s^{2}}}\theta_{ref}+\frac{\left(1-{\tilde{G}}_{a}C_{f}\right)}{1+C_{a}G_{a}+(G_{a}-{\tilde{G}}_{a})C_{f}+C_{v}C_{a}G_{a}\frac{1}{s}+C_{p}C_{v}C_{a}G_{a}\frac{1}{s^{2}}}\theta_{d}$$

As $\theta_{ref}=0$, the disturbance suppression of the system with MFC-DOC method can be given as follows:

(11) $$E_{MFC-DOC}=\frac{\theta }{\theta_{d}}=\frac{1-{\tilde{G}}_{a}C_{f}}{1+C_{a}G_{a}+(G_{a}-{\tilde{G}}_{a})C_{f}+C_{v}C_{a}G_{a}\frac{1}{s}+C_{p}C_{v}C_{a}G_{a}\frac{1}{s^{2}}}$$

Hence, the performance of MFC-DOC depends on the entire three loops controllers as well as the DOC structure.

## 3. Performance Analysis

From the above derivation, the disturbance rejection characteristics of the MFC and the MFC enhanced by DOC are given clearly.

The disturbance rejection characteristics of the MFC can be presented as follows:

(12) $$E_{MFC}=\frac{1}{1+C_{a}G_{a}+C_{v}C_{a}G_{a}\frac{1}{s}+C_{p}C_{v}C_{a}G_{a}\frac{1}{s^{2}}}$$

And the disturbance rejection characteristics of the MFC-DOC can be presented as follows:

(13) $$E_{MFC-DOC}=\frac{1-{\tilde{G}}_{a}C_{f}}{1+C_{a}G_{a}+(G_{a}-{\tilde{G}}_{a})C_{f}+C_{v}C_{a}G_{a}\frac{1}{s}+C_{p}C_{v}C_{a}G_{a}\frac{1}{s^{2}}}$$

where $G_{a}$ is the controlled plant, while ${\tilde{G}}_{a}$ is the approximate model of the controlled plant, which can be obtained by fitting the measured acceleration transfer function. By a spectral analyzer, the acceleration response of the FSM can be obtained in a high accuracy. Thus, we can easily get ${\tilde{G}}_{a}\approx G_{a}$, and it is clear that:

(14) $$(G_{a}-{\tilde{G}}_{a})C_{f}\approx 0$$

So, the denominators of $E_{MFC}$ and $E_{MFC-DOC}$ are almost equal, moreover, the denominators show the stability of the system, which means that the introduction of DOC method has an almost minimal effect on the stability of system. Spontaneously, when comparing with MFC, the disturbance rejection characteristics of MFC-DOC can be simplified as follows:

(15) $${\hat{E}}_{MFC-DOC}=1-{\tilde{G}}_{a}C_{f}$$

According to Equation (15), the disturbance compensation controller $C_{f}$ is of great concern for the effect of DOC, and a zero error remain will be achieved in theory if the controller is designed to an ideal controller as follows:

(16) $$C_{f}≅{\tilde{G}}_{a}{}^{-1}$$

(17) $${\hat{E}}_{MFC-DOC}\to 0$$

The ideal DOC controller is the inverse transfer function of ${\tilde{G}}_{a}$, which can compensate disturbances in all frequencies in theory. As depicted in Figure 3, the input of $C_{f}$ is the estimate of disturbance force and the purpose of $C_{f}$ is to convert the estimated disturbance force into the driver input, by which the disturbance compensation can be completed. After getting ${\tilde{G}}_{a}$, the ability of disturbance compensation depends primarily on the $C_{f}$ design. The ideal DOC controller of FSM can be presented as follows:

(18) $$C_{f\_ideal}=\frac{\frac{s^{2}}{\varpi_{n}{}^{2}}+\frac{2\xi }{\varpi_{n}}s+1}{K}\cdot \frac{T_{e}s+1}{s^{2}}$$

**Figure 4:**
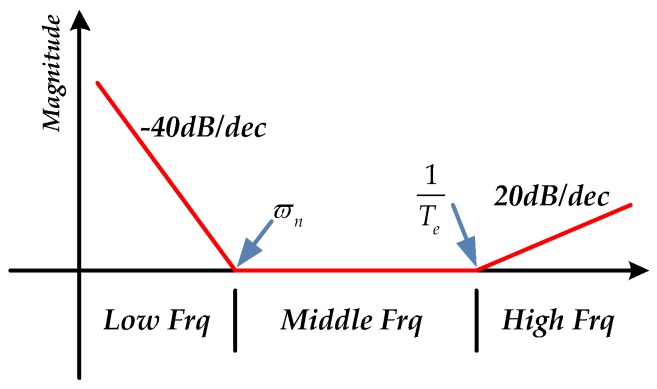
The diagrammatic sketch of the ideal disturbance observation and compensation (DOC) controller in frequency domain.

The open loop natural frequency of FSM $\varpi_{n}$ is approximately between several Hz to tens of Hz, and the damping factor ξ is much smaller than 1 [1,21]. Due to the high sampling frequency in FSM system, the electrical lag factor $T_{e}$ is also much smaller than 1. Thus, it is easy to depict the diagrammatic sketch of the ideal DOC controller in frequency domain as shown in Figure 4.

The full frequency range is divided into three parts in Figure 4, including low frequency, middle frequency and high frequency. Due to the double integration $s^{2}$ and the second-order element, the DOC controller decreases in a slope to −40 dB at low frequency and remains constant at middle frequency. Because of the $T_{e}s+1$ element, it rises in a slope to 20 dB at high frequency. However, because of the low sensitivity of accelerometers at low frequency, the right estimated disturbance force is partly submerged in the noise, which causes the DOC controller to become saturated as a result of the quadratic integration influence. At high frequency, the disturbance effect is mostly restrained by the passive disturbance suppression of FSM platform. As the DOC controller can be regarded as a proportional controller at middle frequency, it is easy to realize it by a low-pass filter. Thus, to take advantage of the middle frequency, the DOC controller is designed in Equation (19):

(19) $$C_{f}=\frac{K_{f}(T_{e}s+1)}{s^{2}+2\xi_{f}w_{f}s+w_{f}{}^{2}}$$

where $T_{e}s+1$ is used to compensate phase lag. The second-order resonance element is used to filter the high-frequency noise of accelerometers and compensate the quadratic integration partly. Substituting Equations (1) and (19) into Equation (15), the disturbance rejection transfer function of MFC-DOC is presented as follows:

(20) $$\begin{array}{l} {\hat{E}}_{MFC-DOC}=1-{\tilde{G}}_{a}C_{f}=1-\frac{KK_{f}\varpi_{n}{}^{2}s^{2}}{\left(s^{2}+2\xi \varpi_{n}s+\varpi_{n}{}^{2})(s^{2}+2\xi_{f}w_{f}s+w_{f}{}^{2}\right)}\\ =\frac{\frac{s^{4}}{\varpi_{n}{}^{2}w_{f}{}^{2}}+2(\frac{\xi }{\varpi_{n}{}^{2}w_{f}}+\frac{\xi_{f}}{\varpi_{n}w_{f}{}^{2}})s^{3}+(\frac{1}{\varpi_{n}{}^{2}}+\frac{4\xi \xi_{f}}{\varpi_{n}w_{f}}+\frac{1}{w_{f}{}^{2}}-\frac{KK_{f}}{w_{f}{}^{2}})s^{2}+2(\frac{\xi }{\varpi_{n}}+\frac{\xi_{f}}{w_{f}})s+1}{\frac{s^{4}}{\varpi_{n}{}^{2}w_{f}{}^{2}}+2(\frac{\xi }{\varpi_{n}{}^{2}w_{f}}+\frac{\xi_{f}}{\varpi_{n}w_{f}{}^{2}})s^{3}+(\frac{1}{\varpi_{n}{}^{2}}+\frac{4\xi \xi_{f}}{\varpi_{n}w_{f}}+\frac{1}{w_{f}{}^{2}})s^{2}+2(\frac{\xi }{\varpi_{n}}+\frac{\xi_{f}}{w_{f}})s+1} \end{array}$$

The damping factor $\xi_{f}$ is much smaller than 1. The designed value of $w_{f}$ should be much bigger than $\varpi_{n}$, otherwise the bandwidth of the controller will be too low. Thus, the ${\hat{E}}_{MFC-DOC}$ can be simplified as follows:

(21) $${\hat{E}}_{MFC-DOC}\approx \frac{(\frac{1}{\varpi_{n}{}^{2}}-\frac{KK_{f}}{w_{f}{}^{2}})s^{2}+2(\frac{\xi }{\varpi_{n}}+\frac{\xi_{f}}{w_{f}})s+1}{\frac{s^{2}}{\varpi_{n}{}^{2}}+2(\frac{\xi }{\varpi_{n}}+\frac{\xi_{f}}{w_{f}})s+1}$$

**Figure 5:**
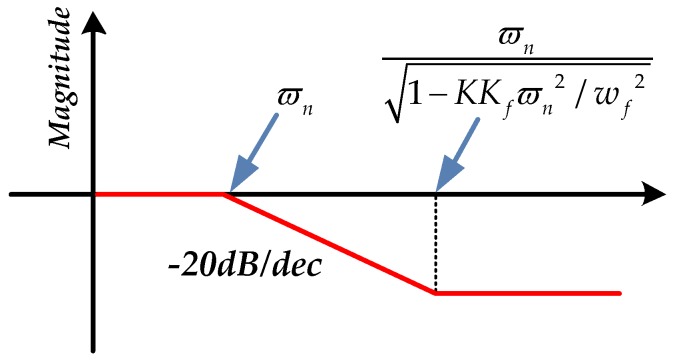
The diagrammatic sketch of the lead-lag corrector.

In Equation (21), the disturbance rejection transfer function of MFC-DOC can be designed into a lead-lag corrector form, the diagrammatic sketch of which is depicted in Figure 5.

As is shown in Figure 5, the compensation at low frequency could be neglected because of the low sensitivity of accelerometers at low frequency. But when the FSM system is suffering middle or high frequency disturbance, it substantially improves the disturbance rejection ability. So without doubt, there will be:

(22) $$E_{MFC-DOC}<E_{MFC}\ll 1$$

It indicates that depending on the lead-lag controller design, the MFC-DOC method has a stronger disturbance rejection ability than the only MFC method; in other words, the system will suffer less influence from the outer disturbance and the remainder error of LOS reduces further.

With MFC-DOC, the sensitivity function of FSM becomes:

(23) $$\begin{array}{ll} S_{MFC-DOC} & =\frac{\left(\frac{(G_{a}+\Delta G_{a})C_{a}C_{v}}{1+(G_{a}+\Delta G_{a})C_{a}+[(G_{a}+\Delta G_{a})-{\tilde{G}}_{a}]C_{f}}\cdot \frac{1}{s}-\frac{G_{a}C_{a}C_{v}}{1+G_{a}C_{a}+(G_{a}-{\tilde{G}}_{a})C_{f}}\cdot \frac{1}{s}\right)/\left(\frac{G_{a}C_{a}C_{v}}{1+G_{a}C_{a}+(G_{a}-{\tilde{G}}_{a})C_{f}}\cdot \frac{1}{s}\right)}{\Delta G_{a}/G_{a}}\\ & =\frac{1-{\tilde{G}}_{a}C_{f}}{1+(G_{a}+\Delta G_{a})C_{a}+[(G_{a}+\Delta G_{a})-{\tilde{G}}_{a}]C_{f}}\\ & \approx \frac{1-{\tilde{G}}_{a}C_{f}}{1+G_{a}C_{a}+(G_{a}-{\tilde{G}}_{a})C_{f}}\\ & \approx \frac{1-{\tilde{G}}_{a}C_{f}}{1+G_{a}C_{a}} \end{array}$$

According to the previous demonstration, if the DOC controller is designed as Equation (19), $S_{MFC-DOC}<S_{MFC}$ can be obtained [9], which means that the introduction of DOC can also improve the robustness of the traditional MFC system. If the controlled model of the system changes substantially, the stability of the velocity loop with DOC will suffer less than that with MFC.

## 4. Controller Design

**Figure 6:**
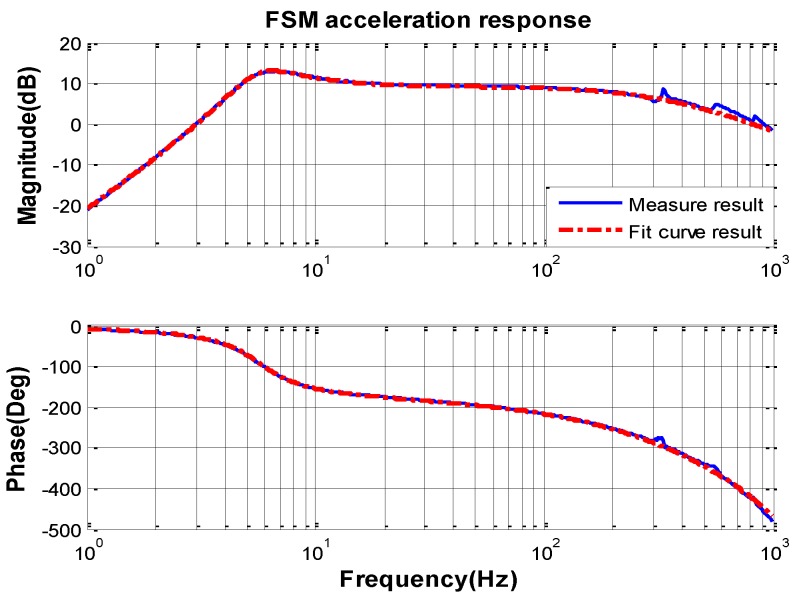
The opened loop characteristic of the FSM acceleration response.

The opened loop characteristic of FSM acceleration response measured by spectral analyzer from 1 Hz to 1000 Hz is shown in Figure 6.

Because of the high bandwidth of the MEMS accelerometer KXR94-2050 (Kionix, New York, NY, USA) [24], the frequency characteristic can be measured to 1000 Hz. By the curve-fitting method and transfer function identification, the mathematic transfer function model of the FSM acceleration response can be obtained as follows:

(24) $${\tilde{G}}_{a}=\frac{0.00225}{0.00078s^{2}+0.019s+1}\cdot \frac{s^{2}}{0.0005s+1}$$

**Figure 7:**
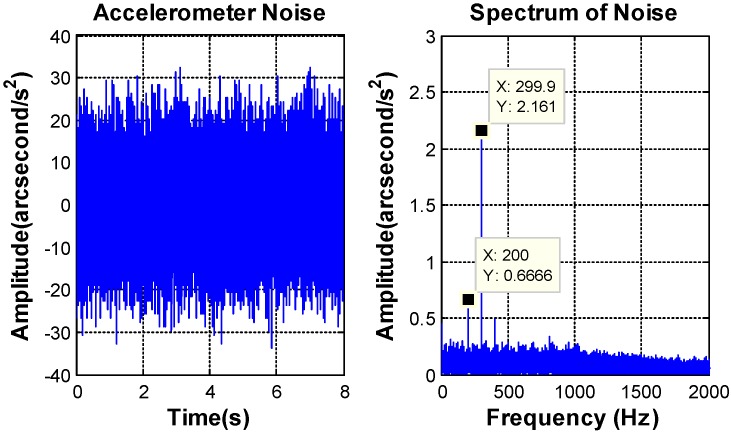
MEMS accelerometer noise characteristics.

It is obvious that the open loop natural frequency of FSM system $\varpi_{n}$ is about 6 Hz and the damping factor ξ is about 0.057. According to the previous analysis, the DOC controller can be designed as Equation (23). The natural frequency $w_{f}$, which decides the bandwidth of the filter is chosen by the characteristics of the sensors noise. The bandwidth of the accelerometer is above 800 Hz and the characteristics of the accelerometer noise are depicted in Figure 7.

**Figure 8:**
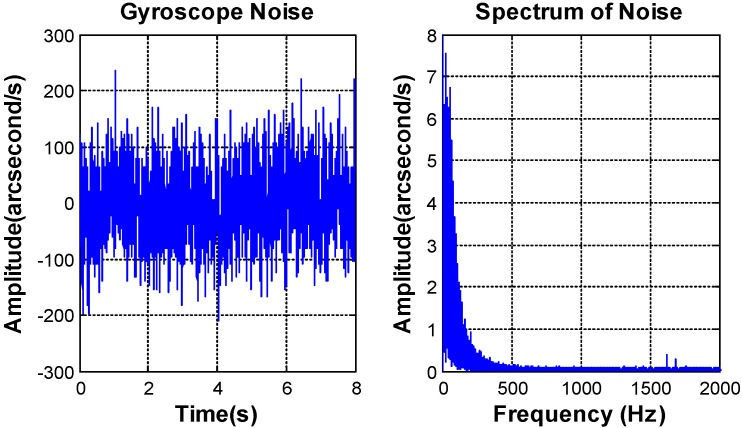
Micro-electro-mechanical system (MEMS) gyroscope noise characteristics.

The peak value of the MEMS accelerometer transformed noise is equal to 0.0097°/s^2^, and the RMS value is about 0.0022°/s^2^. Besides some “spikes”, the amplitude value of amplitude-frequency curve is smooth and the “spikes” are some high frequency noise, which could be filter out by the DOC controller. Considering the noise and the lag of filter, the bandwidth of DOC controller is designed at 40 Hz. Figure 8 shows the characteristics of the MEMS gyroscope noise.

The bandwidth of the MEMS gyroscope SCR1100-D02 (Murata Manufacturing, Nagaokakyo, Kyoto) [25] is less than 100 Hz. The noise mainly focuses on the frequencies ranging from 0 to 200 Hz. The peak value of the MEMS gyroscope noise is equal to 0.069°/s^2^, and the RMS value is about 0.016°/s^2^, which satisfy the requirement of the control system.

Thus, the DOC controller can be designed as follows:

(25) $$C_{f}=\frac{27161\times (0.0005s+1)}{s^{2}+355.4s+63165}$$

where the damping factor $\xi_{f}=0.707$ and the natural frequency $w_{f}=40\;\mathrm{Hz}=80\pi \;\mathrm{rad}/s$, which are both configured by the noise of sensors. To avoid the inaccurate control in acceleration closed loop, the acceleration controller is designed as follows [15]:

(26) $$C_{a}=\frac{260}{s}\times \frac{0.0011s+1}{0.00092s+1}$$

With the completion of loop acceleration, the model of the FSM has been simplified. Therefore, the traditional PI controller can meet the velocity closed-loop and also the position closed-loop [15].

## 5. Experimental Verification

**Figure 9:**
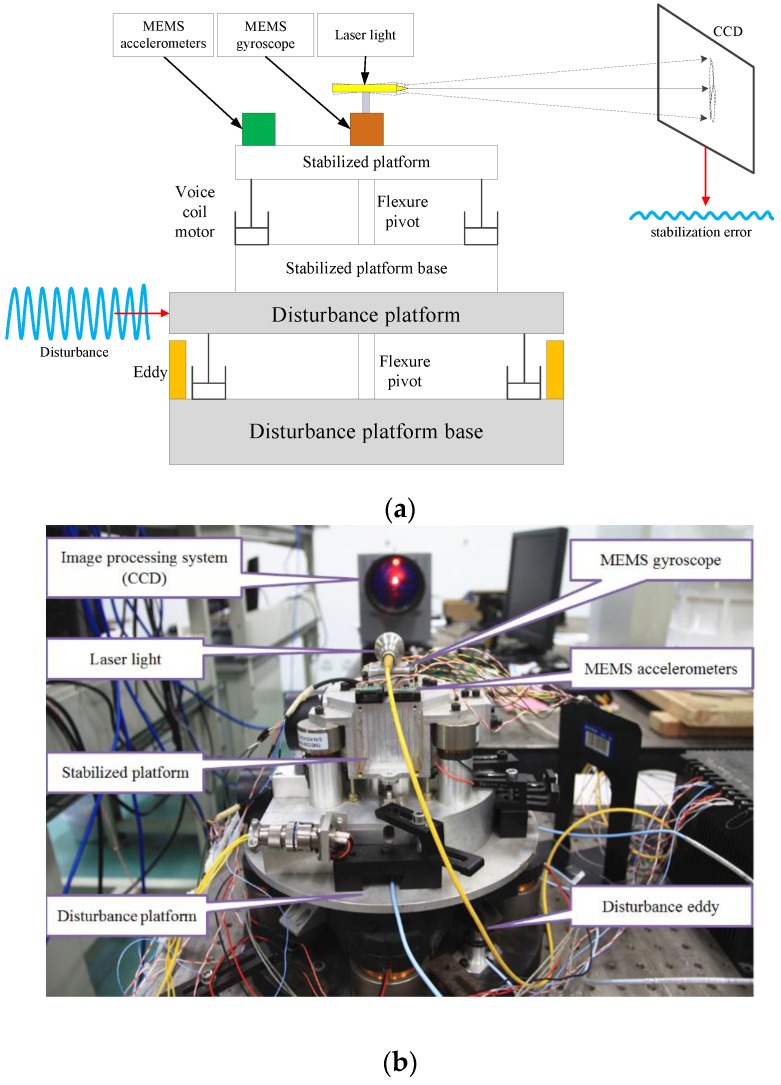
Experimental apparatus. (**a**) Principle of apparatus; and (**b**) Prototype of apparatus.

As shown in Figure 9a, an apparatus constructed by two superimposed FSM systems is used to test and verify the previous analysis. One is used to stabilize the LOS; the other is used to simulate disturbance. The stabilized platform is mounted on the disturbance platform and the laser light is fastened on the top of stabilized platform directly as a reference of LOS. Both of the platforms are driven by the voice coil motors. The eddy sensors in the disturbance platform are used to measure the disturbance input of the stabilized platform and the CCD is used to obtain the stabilization error. Four MEMS linear accelerometers and one MEMS gyroscope are set on the stabilized platform to measure the angular acceleration and angular velocity, respectively. In the experiment, all of the MEMS inertial sensors and the disturbance eddies are at 5000 Hz sampling frequency, moreover, the CCD has only 100 Hz working frequency and 20 ms (two frames) time delay in addition.

**Figure 10:**
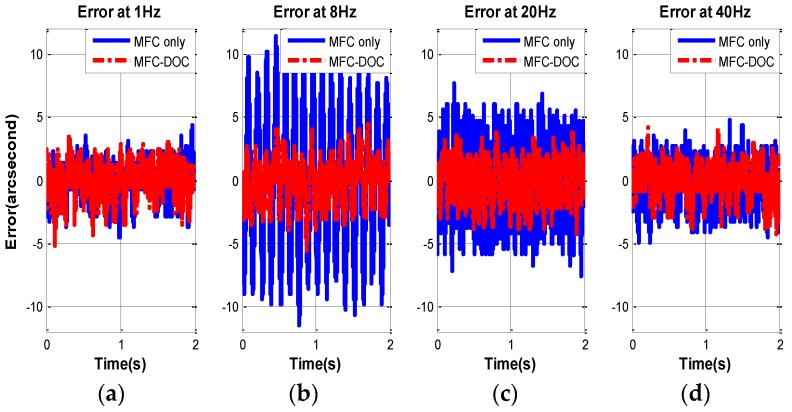
(**a**) Line-of-sight (LOS) error at 1 Hz disturbance; (**b**) LOS error at 8 Hz disturbance; (**c**) LOS error at 20 Hz disturbance; and (**d**) LOS error at 40 Hz disturbance.

The disturbance suppression of FSM system is measured when the stabilized platform control is closed and the disturbance platform is opened. Then, four experiments in different frequency disturbance are compared as follows, and the error of MFC and MFC-DOC are given in Figure 10 and Table 1.

It is obvious that the disturbance suppression performance of MFC-DOC is almost equal to the MFC method at 1 Hz; however, it is improved heavily at 8 Hz and 20 Hz. Due to the passive disturbance suppression characteristics of the FSM platform, the disturbance effect has been mostly restrained, thus, it is the improvement at above 40 Hz is unapparent.

**Figure 11:**
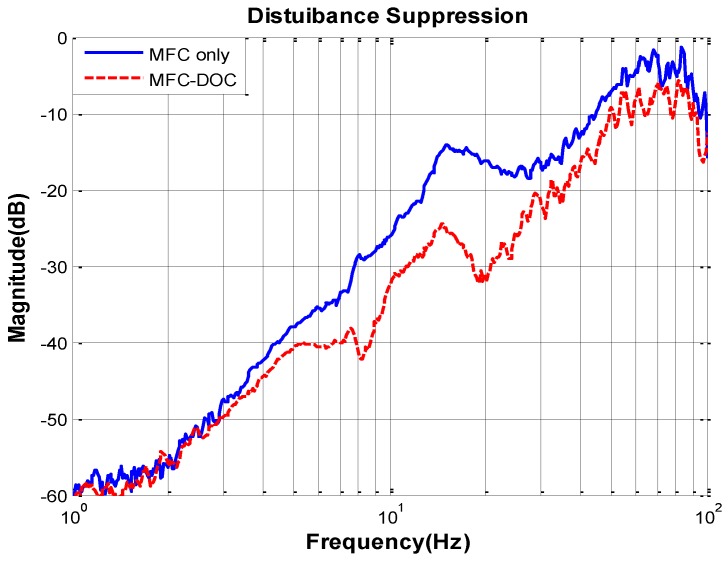
Disturbance suppression characteristics.

Simultaneously, the total disturbance suppression characteristic of the two methods, shown in Figure 11, can be obtained. It can be clearly seen that the disturbance suppression of the FSM system has been improved heavily by DOC at middle frequency, while it almost has no effect at low frequency, which proves the previous analysis.

## 6. Conclusions

The disturbance rejection of LOS is the main purpose of the stabilization of the FSM control system. MEMS inertial sensors have been tried to use in the FSM system because of their relatively small size, low weight and low power consumption. In order to enhance the stabilization performance, the MFC is recommended to raise the stiffness of the FSM system and make stronger disturbance rejection. In this paper, to make further improvement of the stabilization performance, the optimization for MFC with DOC is proposed. The structure of this method is discussed and compared with only the MFC method, which has proved its advantages in theory. Simultaneously, the analysis of stabilization accuracy of this method is presented by contrasting experiments. A series of comparative experimental results show that the disturbance suppression performance of the FSM control system based on the MEMS inertial sensors can be effectively improved by the proposed method.

Future work will concentrate on improving the error attenuation performance of the LOS at low frequency. The use of gyroscopes may be an effective method to estimate the low frequency disturbance, which will be our next work. The sensor fusion of accelerometers and gyroscopes can also be under review.

## Figures and Tables

**Table 1:** The detailed circumstances of stabilization error comparison.

Disturbance Frequency	RMS ^1^ (’’)		Max Peak (’’)	
	MFC	MFC-DOC	MFC	MFC-DOC
**1 Hz**	1.0252	1.0241	4.8598	5.1072
**8 Hz**	5.9126	1.4516	12.2017	4.3802
**20 Hz**	3.0473	1.4623	7.6593	3.7520
**40 Hz**	1.4017	1.0454	4.7773	4.5171

^1^ RMS stands for the Root Mean Square of the error.
